# Work ability 8 years after breast cancer: exploring the role of social support in a nation-wide survey

**DOI:** 10.1007/s10549-022-06599-z

**Published:** 2022-04-21

**Authors:** K. Vandraas, R. S. Falk, S. K. H. Bøhn, C. Kiserud, H. C. Lie, S. K. Smedsland, M. Ewertz, S. Dahl, M. Brekke, K. V. Reinertsen

**Affiliations:** 1 National Advisory Unit of Late Effects after Cancer Treatment, Department of Oncology, Oslo University Hospital, Oslo, Norway; 2 Research Support Services, Oslo Centre for Biostatistics and Epidemiology, Oslo University Hospital, Oslo, Norway; 3 Department of Behavioral Medicine, University of Oslo, Oslo, Norway; 4 Oncology Research Unit, Institute of Clinical Research, University of Southern Denmark, Odense, Denmark; 5 Department of Oncology, Oslo University Hospital, Oslo, Norway; 6 Department of General Practice, Institute of Health and Society, University of Oslo, Oslo, Norway

**Keywords:** Work ability, Breast cancer, Fatigue, Social support

## Abstract

**Introduction:**

As the 5-year survival rate after breast cancer in Norway is 92%, the population of breast cancer survivors (BCSs) is increasing. Knowledge of work ability in this population is scarce. In a population-based cohort of BCSs, we explored work ability 8 years after diagnosis and the association between work ability and social support, and cancer-related variables including late effects and lifestyle factors.

**Methods:**

In 2019, all Norwegian women < 59 years when diagnosed with stage I–III breast cancer in 2011 or 2012, were identified by the Cancer Registry of Norway and invited to participate in a survey on work life experiences. Work ability was assessed using the Work Ability Index (scale 0–10). Factors associated with excellent work ability (score ≥ 9) were identified using univariate and multivariate logistic regression analyses, and adjusted for socioeconomic-, health- and cancer-related variables.

**Results:**

Of the 1951 eligible BCSs, 1007 (52.8%) responded. After excluding survivors with relapse (*n* = 1), missing information on work ability score (*n* = 49), or work status (*n* = 31), the final sample comprised 926 BCSs within working age at survey (< 67 years).

Mean age at survey was 56 years and 8 years (SD 0.7) had passed since diagnosis. Work ability had been reduced from 8.9 (SD 2.3) at diagnosis to 6.3 (SD 3.1). One in three BCSs reported poor work ability (WAS ≤ 5), and seven out of ten reported that their physical work ability had been reduced due to cancer. Social support from colleagues during cancer therapy was associated with excellent work ability, which was not observed for social support provided by supervisors or the general practitioner. Cognitive impairment and fatigue were inversely associated with work ability. None of the cancer-related variables, including treatment, were associated with work ability 8 years after diagnosis.

**Conclusion:**

In this population-based sample, one in three BCSs reported poor work ability 8 years after diagnosis. Collegial social support during cancer therapy appears to be a protective factor for sustained work ability, whilst survivors struggling with fatigue and cognitive impairments may represent a particularly vulnerable group for reduced work ability.

## Introduction

In 2020, 3424 women were diagnosed with breast cancer in Norway, corresponding to a cumulative risk of developing breast cancer before the age of 80 years of 8.7%. Incidence has doubled during the last five decades, and the 5-year relative survival rate for all stages has reached 92.1%. Consequently, the number of breast cancer survivors (BCSs) is increasing with a population close to 53,000 in Norway in 2020 [1].

Work ability may be defined as an individual’s physical, psychological, and social resources for participation in any kind of paid work or self-employment [2]. Work ability is a key issue for BCSs given the importance of work participation on identity, quality of life, financial security, and social relations [3, 4] and its close relation to work status [5]. Research on work outcomes in BCSs has mainly focused on the early period after breast cancer therapy, and show that the majority of BCSs successfully return to work within 2 years post-diagnosis [6–9]. However, work ability beyond the first 5 years of breast cancer survivorship has received less attention, except for a few studies reporting poorer work ability among early-stage, long-term BCSs compared to healthy, age-matched controls [2, 10]. This later phase of survivorship may be challenging given the high risk of treatment-related late effects, including fatigue, cognitive dysfunction, pain, sleep problems, depression, and fear of cancer recurrence [11, 12], which may represent barriers to sustained employment [13]. Increased fatigue have been shown to predict impaired return to work during the first 18 months post-diagnosis [14], but knowledge of late effects and work ability beyond this point is scarce.

Social support is not a clearly defined term, but may encompass emotional (for instance expression of positive feelings), practical (for instance provision of material aids), and informational aspects (for instance offering advice and guidance) [15]. According to the ‘buffer hypothesis’ social support may protect individuals from the negative consequences of life stressors by increasing their access to coping resources [16]. Social support in general is shown to reduce the effects of psychological and physical distress [17, 18], and at the work place increase productivity and workers’ well-being [19]. In a cancer-specific context and according to the ‘buffer hypothesis’, social support may mediate the consequences of late effects from cancer treatment, such as depression and anxiety [20] and increase over-all quality of life [21]. In addition to family and close friends, colleagues, supervisors, and the general practitioners (GPs) represent important sources of social support for work-related outcomes [22]. Social support from these providers is reported to have a beneficial effect on cancer survivors returning to the work place [23] but concerning long-term work life the role of social support is less clear. In a study on 3–5 year survivors of different cancers, 39% of women hoped for more work place support than they had received, and they reported higher supportive needs than the male cancer survivors [18].

Despite its relevance and importance, there is a substantial gap in knowledge concerning work ability beyond the first 5-year period for BCSs, and the role of social support and late effects. The objective of this study was therefore to explore these associations in a population-based cohort of BCSs 8 years after diagnosis.

## Materials and methods

### Study population

The **S**urvivorship **W**ork and S**e**xual H**e**al**t**h (SWEET)-study was a nation-wide survey of Norwegian long-term BCSs performed in 2019. The Cancer Registry of Norway identified all survivors of stage I–III breast cancer diagnosed in 2011 or 2012 at the age of 20–65 years. Among the 2803 survivors who were invited, 1951 women were below 59 years at diagnosis (corresponding to younger than the legal retirement age in Norway (< 67 years) at survey 8 years later), of whom 1007 survivors (52.8%) responded after one reminder. After excluding survivors with cancer relapse (*n* = 1), missing information on work ability score (*n* = 49), or work status (*n* = 31), the final sample for this sub-study consisted of 926 BCSs.

### Attrition analyses

Compared to responders, non-responders were on average 1.3 years older at diagnosis, had lower tumor proliferation markers (mean Ki67-value of 27 versus 31), and an additionally 4% were Her-2 negative (85% versus 81%) compared to the responders. The groups did not differ according to tumor size, nodal involvement, hormone receptor status, or type of surgery (results not shown).

### Primary outcome

The Work Ability Index (WAI) includes seven items, is validated and the most commonly used self-report instrument for measuring work ability [24]. In line with previous studies on work ability among cancer survivors [5, 6], we applied three of these items as this has been reported to yield comparable validity compared to using the whole WAI [25] while being less time-consuming.

*Item one of the WAI*, the Work Ability Score (WAS), has been identified as a valid single-indicator of work ability [26] and was therefore applied as the dependent variable in the regression analyses. Using the WAS survivors rated their current work ability on a scale from 0 to 10, where 10 reflect highest possible work ability and zero implies extinguished work ability. Scores were categorized into four categories; poor, moderate, good, and excellent (Table 2). *In item two of the WAI*, survivors were asked to describe their current work ability with respect to the physical (question 1) and the mental (question 2) demands of their work. Response categories ranged from very bad (1) to very good (5). *In item three*, survivors were asked to rate to what extent their physical (question 1) and mental (question 2) work ability had been affected by cancer. Response categories ranged from a lot (1) to not at all (5). More details on the WAI are displayed in Table 2.

### Explanatory variables

#### Clinical variables

Age at diagnosis and information on surgical treatment were supplied by the Cancer Registry of Norway. All other variables, including systemic cancer therapies, were based on self-report. Use of primary health care services was explored by asking survivors to report the number of visits to the general practitioner (GP) during the last 12 months.

#### Socioeconomic variables

Age at survey, living arrangements, educational attainment, and work status at diagnosis and at survey were based on self-report (Table 1). Survivors rated their work ability at time of diagnosis on the same scale as used for the WAS, and these scores were used to assess change in work ability from diagnosis to survey.

**Table 1:** Characteristics of 926 Norwegian breast cancer survivors at diagnosis (2011/2012) and at survey (2019)

Socioeconomic variables*	
Age at survey in years, mean (SD)	55.8 (6.9)
Living with partner, *n* (%)	705 (76.1)
Education, n* (%)	
Long (> 12 y)	536 (57.9)
Short (≤ 12 y)	385 (41.6)
Number of visits to the general practitioner (GP) last 12 months	3.9 (4.7)
Work-related variables, at diagnosis, *n* (%)	
Paid work**	827 (89.3)
Disability pension	58 (6.3)
Retired or other work statuses	41 (4.4)
Self-reported work ability (score range of 0–10), mean (SD)	8.9 (2.3)
Work-related variables, at survey, *n* (%)	
Paid work**	547 (59.1)
Disability pension	289 (31.2)
Retired or other work statuses***	90 (9.7)
Work ability score (WAS, score range of 0–10), mean (SD)	6.3 (3.1)
Social support at the work place and from the general practitioner, mean (SD)	
Demand-control-support questionnaire (DCSQ) items 1–6 (score range of 6–24)	9.9 (3.1)
Structural–functional-support-scale (SFSS) (score range of 4–20 per provider)	
Supervisor support (item 1–4)	12.3(4.2)
Colleague support (item 5–8)	12.4 (3.7)
General Practitioner support (item 9–12)	10.8 (4.4)
Lifestyle, *n* (%)	
Physically active****	401 (43.3)
Overweight (Body Mass Index > 25 kg/m^2^)	489 (52.8)
Clinical variables	
Age at diagnosis in years, mean (SD)	47.8 (7.0)
Years since diagnosis, mean (SD)	8.0 (0.7)
Treatment, *n* (%)	
Radiation	733 (79.2)
Breast conserving therapy	496 (53.6)
Chemotherapy	732 (79.0)
Chemo- and endocrine therapy	567 (61.0)
Health related quality of life	
Sleep problems, *n* (%)	427 (46.1)
Neuropathy, *n* (%)	186 (20.1)
EORTC-QLQ-C30/BR-23 (score range of 0–100), mean (SD)*****	
Cognitive function	71.5 (25.3)
Fatigue	40.2 (27.6)
Pain intensity	27.6 (29.0)
Arm symptoms	22.4 (25.3)
Breast symptoms	16.8 (19.6)
Depression (score range of 0–27), mean (SD)	6.4 (4.6)
Fear of cancer recurrence (score range of 0–40), mean (SD)	12.0 (8.7)
Anxiety (score range of 0–21), mean (SD)	4.1 (3.6)

*SD* standard deviation, *EORTC* European Organization for Research and Treatment of cancer

*Sums may not does add up to 926 due to missing values

**Paid work = part time work, full time work, self-employment and sick-leave

***other statues = included job seeker, temporarily laid off, work allowance, education or military service and homemaker

****Physically active =  ≥ 150 min of moderately intense- or ≥ 75 min of vigorously intense weekly activity

*****High score = high symptom intensity (pain, fatigue, arm/breast symptoms) or high cognitive function

#### Social support

Received cancer-specific social support was measured using 12 items (scale A) from the Structural and Functional Support Scale (SFSS) [27, 28]. In the survey, survivors were asked to evaluate to what extent they had received support from three selected support providers; their supervisors, colleagues, and the GP during cancer therapy, by scoring each item from 1 (not at all) to 5 (a lot) [18] (Table 1). We dichotomized the sum score into high and low received support (Table 2). The SFSS has good psychometric properties [28]. Cronbach’s alpha was 0.90 for supervisor support, 0.88 for collegial support, and 0.89 for GP support. Current social support at the workplace was described using the support sub-scale from the Demand Control Support Questionnaire (DCSQ) [29] (Table 1). Cronbach’s alpha was 0.90 for the DCSQ.

**Table 2:** Items form the Work Ability Index including the Work Ability Score, in total and according to response category, among 926 breast cancer survivors 8 years after diagnosis

Item 1 Current work ability compared to life-time best (0–10)	
Q*: Assume that your work ability at its best has a value of ten points.	
How many points would you give your current work ability?	*n* (%)
Excellent (10 points) WAS category 4	151 (16.3)
Good (8–9 points)	264 (28.5)
Moderate (6–7 points)	206 (22.2)
Poor (0–5 points) WAS category 1	305 (32.9)
Total	926 (100)*
Item 2 Work ability in relation to the demands of the job (1–5)	
Q 1: How do you rate your current work ability with respect to the physical demands of your work?	
Very good (score 5)	313 (33.8)
Pretty good (score 4)	245 (26.5)
Fairly good (score 3)	185 (20.0)
Quite bad (score 2	73 (7.9)
Very bad (score 1)	66 (7.1)
Total	882 (95.2)*
Q 2: How do you rate your current work ability with respect to the mental demands of your work?	
Very good (score 5)	313 (33.8)
Pretty good (score 4)	255 (27.5)
Fairly good (score 3)	190 (20.5)
Quite bad (score 2)	78 (8.4)
Very bad (score 1)	43 (4.6)
Total	879 (94.9)*
Item 3 Estimated work impairment due to diseases (1–5)	
Q 1: Has your physical work ability been reduced due to cancer?	
Not at all (score 5)	224 (24.2)
A little bit (score 4)	166 (17.9)
To some extent (score 3)	253 (27.3)
Quite a bit (score 2)	144 (15.6)
A lot (score 1)	101 (10.9)
Total	888 (95.8)*
Q 2: Has your mental work ability been reduced due to cancer?	
Not at all (score 5)	260 (28.1)
A little bit (score 4)	238 (25.7)
To some extent (score 3)	217 (23.4)
Quite a bit (score 2)	120 (13.0)
A lot (score 1)	55 (5.9)
Total	890 (96.1)*

*Q* question, *WAS* work ability score

*Total sample

#### Life style variables

Overweight was defined as Body Mass Index > 25 kg/m^2^ [30] and physical activity according to a modified version of the Godin Leisure Time Exercise Questionnaire [31] (Table 1).

#### Health related quality of life

Sleep problems were defined according to The Trøndelag Health Study [32], neuropathy was assessed using the scale of chemotherapy induced long-term neurotoxicity (SCIN) [33], while cognitive function, pain, and fatigue were assessed using subscales from the European Organization for Research and Treatment of Cancer Quality of Life Questionnaire (EORTC QLQ-C30) version 3 [34], and arm and breast symptoms by the breast cancer-specific module (EORTC BR 23)[35] (Table 1). Cronbach’s alphas were 0.72 for cognitive functioning, 0.87 for pain, 0.89 for fatigue, 0.79 for arm-, and 0.78 for breast symptoms.

#### Depression, anxiety, and fear of cancer recurrence

Depressive symptoms were explored using the Patient Health Questionnaire (PHQ-9) [36]. We imputed values for the PHQ-9 by substituting missing values with mean values if no more than two items were missing. Cronbach’s alpha for the PHQ-9 was 0.80. Anxiety symptoms were evaluated using the General Anxiety Disorder 7-item tool (GAD-7) [37]. We imputed values for the GAD-7 by substituting missing values with mean values when more than 50% of items had been answered. Cronbach’s alpha for GAD-7 was 0.90. Fear of cancer recurrence was assessed using four items from the Concern about Recurrence Questionnaire (CARQ) [38]. Cronbach’s alpha for CARQ was 0.70.

### Statistical analyses

Descriptive analyses were performed for the total sample, describing continuous variables using means and standard deviations and categorical variables as numbers and percentages. To describe the relationship between social support during cancer therapy and work ability, we performed cross tabulations between the different items of SFSS for each provider of support and WAS. *p* values from Pearson’s chi-squared test and Cramér’s *V* were reported. To explore factors associated with excellent work ability at survey we applied logistic regression models with WAS as the dependent variable (score 10 vs. score 0–9). Body mass index was considered a continuous variable. Explanatory variables associated with excellent work ability at significance level of ≤ 0.2 in univariable analyses were included in the multivariable model.

Collinearity statistics were performed for sets of variables which theoretically may overlap. This included age at diagnosis, cognitive function, fatigue, depression, anxiety, and fear of cancer recurrence, with highest observed variation inflation factor of 3.3 which is considered acceptable. As fear of cancer recurrence and anxiety has overlapping symptomatology, we chose to exclude anxiety from the final model. Furthermore, as the majority of BCSs who were disabled at time of survey rated poor current WAS, and work status and work ability are closely related, we excluded work status from the regression analyses. Results were presented as odds ratios (OR) with 95% confidence intervals (CI) and accompanying *p* values.

In order to assess the potential risk of non-response bias, the CRN provided data concerning cancer-related variables for the non-responders. Comparisons of responders vs. non-responders were performed by comparing mean values for these selected variables. All analyses were performed using IBM SPSS version 26.0 (SPSS, Chicago, IL).

## Results

Mean age of included BCSs was 55.8 (SD 6.9) years at survey, and 8 years had passed since BC diagnosis (SD 0.7). The majority lived with a partner (76%) and had long education (58%). At diagnosis, 89% were in paid work, while 59% were in paid work at survey, and the proportion of survivors receiving disability pension increased from 6.3 to 31.2% during this time period. Mean WAS at survey was 6.3 (SD 3.1), reduced from 8.9 (SD 2.3) at time of diagnosis. About half of the survivors were overweight (53%) and 57% were physically inactive. Almost 80% had received chemotherapy (Table 1).

Sixteen percent rated their work ability as excellent, while 33% rated their work ability as poor. Close to 45% (44.8%) reported good or excellent work ability. With respect both to the physical and mental demands of their work, 34% rated their work ability as very good. On the other end of the scales, 15% and 13% rated their work ability as quite bad or very bad with regards to the physical and mental demands at work, respectively. Approximately 72% (71.7%) reported that their physical work ability had been reduced due to cancer, while 68% reported that their mental work ability had been reduced due to cancer (Table 2).

Survivors with excellent work ability reported that they had received a high degree of support from supervisors (72%) and from colleagues (79%) during cancer therapy, while 41% reported a high degree of support provided by their GPs. Among survivors with poor work ability, significantly lower proportions (48 and 51%, respectively) reported that they had received a high degree of support from their supervisor and colleagues compared to those with excellent work ability. The association between received social support and WAS was statistically significant for supervisor- and collegial-support (Cramèr’s *V* 0.16–0.19), but not for GP-provided support (Table 3).

**Table 3:** Received social support during cancer therapy from supervisors, colleagues, and the general practitioner according to work ability score category among Norwegian breast cancer survivors 8 years after diagnosis

Received social support	Work ability score category from poor (1) to excellent (4)				*n*	*p* value	Cramér’s *V*
	1 (score 0–5) *N* (%)	2 (score 6–7) *N* (%)	3 (score 8–9) *N* (%)	4 (score 10) *N* (%)			
From supervisor (*n*: 824)						< 0.001	0.16
High*	121 (48.4)	99 (51.0)	138 (56.1)	96 (71.6)	454		
Low**	129 (51.6)	95 (49.0)	108 (43.9)	38 (28.4)	370		
From colleagues (*n*: 827)						< 0.001	0.19
High*	127 (51.0)	106 (55.5)	147 (59.3)	110 (79.1)	490		
Low**	122 (49.0)	85 (44.5)	101 (40.7)	29 (20.9)	337		
From GP (*n*: 852)						0.71	–
High*	119 (45.1)	80 (40.0)	107 (43.0)	57 (41.0)	363		
Low**	145 (54.9)	120 (60.0)	142 (57.0)	82 (59.0)	489		

*High: SFSS sum score 12–20, **Low: SFSS sum score 4–11

In the univariable analyses, positive associations were observed for the following variables: age at survey (OR 1.04, 95% CI: 1.01–1.07), collegial- (OR 1.16, 95% CI 1.10–1.22) and supervisor support during cancer therapy (OR 1.09, 95% CI: 1.04–1.14) and cognitive function (OR 1.06, 95% CI: 1.05–1.08). Increasing BMI was inversely associated with work ability (OR 0.96, 95% CI: 0.92–0.99), as was physical inactivity (OR 0.47, 95% CI: 0.33–0.68), low current support (OR 0.81, 95% CI: 0.76–0.87), receiving chemotherapy (OR 0.35, 95% CI 0.24–0.52), and endocrine treatment (OR 0.65, 95% CI: 0.45–0.93). Furthermore, the presence of all late effects was inversely associated with work ability; sleep problems (OR 0.43, 95% CI:0.29–0.62), neuropathy (OR 0.30, 95% CI:0.16–0.55), fatigue (OR 0.94, 95% CI: 0.93–0.95), pain (OR 0.96, 95% CI: 0.94–0.97), arm symptoms (OR 0.96, 0.94–0.97), breast symptoms (OR 0.96, 95% CI: 0.94–0.97), depression (OR 0.75, 95% CI: 0.70–0.80), and fear of cancer recurrence (OR 0.93, 95% CI:0.91–0.96) (Table 4).

**Table 4:** Factors associated with excellent work ability among Norwegian breast cancer survivors 8 years after diagnosis

	OR	95% CI	OR adj	95% CI	*p* value
Age at survey (years)	1.04	1.01–1.07	1.03	0.98–1.08	0.33
Body Mass Index (kg/m^2^)	0.96	0.92–0.99	1.01	0.94–1.09	0.75
Physically inactive	0.47	0.33–0.68	0.61	0.32–1.18	0.14
Number of visits to the GP	0.69	0.61–0.79	0.85	0.72–1.01	0.07
Support					
Current social support at work (DCQS)	0.81	0.76–0.87	0.98	0.85–1.13	0.78
Cancer-specific social support (SFSS)					
Supervisor support	1.09	1.04–1.14	0.96	0.86–1.07	0.45
Colleague support	**1.16**	**1.10–1.22**	**1.16**	**1.02–1.31**	**0.03**
Cancer-related variables					
Receiving chemotherapy (ref: no)	0.35	0.24–0.52	0.63	0.29–1.36	0.24
Receiving endocrine treatment (ref: no)	0.65	0.45–0.93	0.97	0.48–1.95	0.93
Health related quality of life					
Sleep problems (ref: no)	0.43	0.29–0.62	0.75	0.37–1.50	0.41
Neuropathy (ref: no)	0.30	0.16–0.55	0.92	0.30–2.83	0.88
Fatigue^a^	**0.94**	**0.93–0.95**	**0.96**	**0.94–0.99**	** < 0.01**
Cognitive function^a^	**1.06**	**1.05–1.08**	**1.03**	**1.01–1.05**	**0.02**
Pain intensity^a^	0.96	0.94–0.97	0.99	0.97–1.01	0.35
Arm symptoms^a^	0.96	0.94–0.97	0.98	0.96–1.01	0.15
Breast symptoms^a^	0.96	0.94–0.97	0.99	0.96–1-02	0.49
Depression^b^	0.75	0.70–0.80	1.12	0.97–1.29	0.12
Fear of cancer recurrence^c^	0.93	0.91–0.96	0.99	0.95–1.03	0.55

*DCSQ* demand control support questionnaire, *SFSS* social functional support scale, *OR* odds ratio, *CI* confidence interval, *adj* adjusted, *GP* general practitioner, *ref* reference

^**a**^Scale 0–100

^**b**^Scale 0–27

^**c**^Scale 0–40. Only univariate analyses with significance level of ≤ 0.2, are shown here. Significant adjusted associations are highlighted in bold

After adjustments, collegial support during cancer therapy, but not supervisor support or current social support, remained positively associated with work ability (OR 1.16, 95% CI: 1.02–1.31, *p* value 0.03), as was cognitive function (OR 1.03, 95% CI: 1.01–1.05, *p* value 0.02), while fatigue was inversely associated with work ability (OR 0.96, 95% CI 0.94–0.99, *p* value < 0.01). Cancer-related variables including chemo- and endocrine treatment, work-related variables and other late effects including sleep problems, neuropathy, pain, arm- and breast symptoms, depression, and fear of cancer recurrence, were no longer associated with work ability (Table 4).

## Discussion

This study indicates that BCSs have work-related challenges beyond the first few years after diagnosis. One in three reported poor current work ability 8 years after diagnosis, and approximately 70% reported that their physical or mental work ability had, to some degree, been reduced due to cancer. Receiving collegial support during cancer therapy stands out as an important and lasting protective factor for sustained work ability.

Work ability diminished significantly from time of diagnosis (8.9) until survey 8 years later (6.3). This was also reflected in a significant reduction in work status, where one in four of the BCSs went from being in paid work to receiving disability pension during this time period. A WAS of 6.3 is lower than previously reported for BC survivors. A Danish study of long-term BCSs examined > 5 years after diagnosis, report significantly poorer work ability among BCSs compared to cancer-free controls [10]. In that study, mean WAS was 8.7 among the BCSs compared to 8.9 among controls, i.e., it was still almost three points higher on the 0–10 scale than we report. Also in a Nordic study, comparing work ability among long-term survivors of breast-, prostate-, and testicular cancer to that of the general population, work ability was lower (WAS 8.4) among BCSs compared to cancer-free controls [39]. These studies only included survivors in paid work, and given the close association between work status and work ability, this may account for the relatively high WAS-scores reported in those studies compared to our finding.

A WAS of 6.3 is also lower than reported among survivors of other cancer forms, including males. This is in line with reports that female cancer survivors in general report lower work ability [2], more work-related limitations [40], and higher supportive care needs at work compared to males [18]. In a study on long-term Norwegian prostate cancer survivors, 75% of patients reported good or excellent work ability and mean WAS for the total sample was 8.6 [41]. It remains unknown whether gender is the common denominator here or if there are unique aspects related to BC treatment or survivorship influencing these observations. In a mixed cancer survivor sample including 26% BCSs examined 18 months post-diagnosis, de Boer et al. reported a WAS of 6.7 [6]. Interestingly, they observed that work ability steadily increased during the first 18 months after diagnosis. Our findings indicate that this increase in work ability may come to a halt at some time point during long-term BC survivorship.

Clinical factors related to diagnosis and cancer therapy, identified as strong predictors for the return to work-process [14] had lost their significance after 8 years. None of the treatment- or cancer-related variables were associated with work ability, including chemotherapy. However, two of the most common late effects after BC, fatigue and cognitive impairment, were significantly associated with work ability. This finding is in line with results in the study by Carlsen et al. who identified fatigue as one of the strongest predictors of low work ability among BCSs examined > 5 years post-diagnosis, increasing the risk of low work ability close to eleven times among affected individuals [10]. Furthermore, BCSs in the present study reported higher levels of fatigue and lower cognitive function than reported by age-appropriate Norwegian norms [42], confirming that a substantial proportion of BCSs struggle with these complaints more than 5 years post BC [43, 44]. From a clinical perspective this is important knowledge as it identifies sub-groups of BCSs who may be in need of specific attention during survivorship care.

Collegial support during cancer therapy was identified as the only source of social support associated with excellent work ability. This suggests that the more informal, day-to-day emotional social support characterizing a good working environment may be more important for work ability than informational and practical support provided by supervisors and GPs. Interestingly, the same association was not observed for current social support at the workplace. These findings suggest that how BCSs perceived their social support systems at work during cancer therapy may have long-lasting effects on their work ability. The majority of BCSs rated their GP-provided support as low as lower than the other sources of social support explored here. Among survivors with poor work ability, a larger proportion reported that they had received low support from their GP compared to the support they had received at their workplace. Survivors with excellent work ability reported a high degree of support from both supervisors and colleagues (72–79%), while only 41% of them reported high support from their GPs.

The cross-sectional design of this study does not allow us to conclude whether high collegial support during cancer therapy results in high work ability long after the BC diagnosis, or if having high work ability reduces the need for social support. This relation may also differ between the different support providers. For instance, BCSs must actively seek contact with their GPs to receive GP support. BCSs with high work ability are probably less likely to do so compared to BCSs struggling with work-related issues. Furthermore, this study has shown that poor work ability is associated with fatigue and cognitive challenges. Survivors struggling with these issues may be in closer contact with their GPs than those without these complaints who also probably have higher work ability. Consequently, report on low perceived GP support among those with high work ability may merely reflect less need for and thus contact with this provider, while that of colleagues and supervisors are a more integrated part of their working environment. On the other hand, we cannot rule out the possibility that BCSs with low work ability in fact lack work-related support from their GPs, for instance due to lack of knowledge of late effects after cancer and work life participation.

## Strengths and limitations

The major strength of this study is the quality of the registry data. Survivors were identified by the CRN, which provides close-to complete and highly accurate estimates for the Norwegian cancer population [45]. The results are therefore likely generalizable to BCSs of working age within societies with comparable welfare systems as the Norwegian. Other strengths include sample size and the use of established and validated outcome-measures.

Several limitations need to be addressed, the cross-sectional retrospective design being the most important. Given this study design we cannot disentangle whether social support influences work ability, or the reverse, work ability influences received support and support-seeking behavior. Work ability and most of the explanatory variables included in the regression analyses, including SFSS, were self-reported and may be subject to recall-bias. The response rate in the SWEET-study is considered acceptable for current population-based studies [46]. The risk of selection bias was explored in attrition analyses but only minor differences were observed between responders and non-responders. However, we cannot entirely rule out that selection bias may have been present. Concerning generalizability, the results must be interpreted with caution within more heterogeneous BC populations, especially regarding socioeconomic status. The Norwegian welfare systems offer financial security for those who are unable to work. Thus, reasons for staying within the work force may be different in non-Western populations and may not be directly comparable to our findings. Finally, SFSS measures social support during cancer therapy and consequently we do not have information on present GP support. Work ability and supportive needs may have fluctuated since time of diagnosis.

## Conclusion

One third of BCSs reported low work ability 8 years after diagnosis, and that their work ability had been substantially reduced since time of diagnosis. Collegial support was identified as the most important source of social support with regards to work ability, and underlines the importance of work place support during cancer therapy on long-term work outcomes for BCSs. Future efforts need to focus on improving and extending the supportive role of the GPs concerning vocational guidance for BCSs. BCSs struggling with fatigue and cognitive dysfunction may represent a particularly vulnerable group of BCSs where clinician attention is warranted during follow-up care.

## Data Availability

All data is available at the National Advisory Unit for Late Effects after Cancer Treatment, Department of Oncology, Oslo University Hospital, the Radium Hospital, Oslo, Norway.
